# Development of a mobile application to estimate time of death based on the compound method

**DOI:** 10.1007/s00414-026-03811-3

**Published:** 2026-04-24

**Authors:** Andrea Zirn, Celine Berger, Holger Wittig, Joel Bottoni, Kathrin Gerlach, Eva Scheurer, Claudia Lenz

**Affiliations:** 1 Institute of Forensic Medicine, Department of Biomedical Engineering, University of Basel, Pestalozzistrasse 22, Basel, 4056 Switzerland; 2 Institute of Forensic Medicine, Health Department Basel-Stadt, Basel, Switzerland

**Keywords:** Time of death, Time of death estimation, Mobile application, Compound method, Early postmortem interval, ToD

## Abstract

During legal inspection, the time of death estimation based on the compound method incorporates the early postmortem changes livor, rigor and algor mortis, as well as supravital reactions. Currently, in forensic routine, the time of death estimation is usually performed on-site during the legal inspection. To streamline the procedure and facilitate its practical application, we developed a mobile application called “ToD” allowing the time of death estimation based on the compound method digitally on-site. To provide high performance and an intuitive user interface, the software Flutter was selected as development framework. The calculation engine of the ToD app operates without internet connection and delivers rapid and reliable calculations. Therefore, our ToD application enables forensic pathologists to perform real-time, on-site time of death estimations, replacing cumbersome paper-pencil based calculations.

## Introduction

The compound method by Henssge and Madea is considered the gold standard for narrowing down the time of death in the early postmortem interval. This approach incorporates the temperature-based calculation as well as non-temperature-based criteria concerning lividity, rigor mortis, mechanical and electrical excitability of the skeletal muscle and the pharmacological excitability of the iris [1–5].

Following death, the thermoregulatory processes cease [6]. As a result, the body exchanges energy with its surroundings until it attains thermal equilibrium with the environment [7]. Building on this principle, Henssge established a method for estimating the time of death based on the cooling properties of the body by measuring the core body temperature in conjunction with the environmental temperature, while considering the body weight of the deceased [4].

In addition to temperature-based methods, early postmortem changes such as livor mortis provide important supplementary information. Following cessation of circulation after death, blood flows in the dependent areas of the body under the influence of gravity. The apparent external postmortem discolorations is a consequence of the blood moving into the capillaries of the corium [8]. Initial small spots progressively confluence into larger patches with an increasing postmortem interval [8]. Only in the early stages of postmortem changes, the livor mortis can be completely blanched by thumb pressure and redistributed by repositioning the body which can refine the estimation of the time of death [8, 9].

Rigor mortis is another early postmortem change that develops as adenosine triphosphate (ATP) levels decline, resulting in progressive muscular stiffness. Hereby, the asynchronous stiffening of muscle fibers accounts for both the gradual increase in rigidity and its reappearance following forced joint manipulation [10–12]. Mallach observed that the onset of the rigor mortis manifests within the first seven hours postmortem [9]. Even if disrupted by external force, stiffness can reestablish as additional fibers enter rigor, with full development occurring within the early postmortem period [9, 13].

After death, tissue metabolism does not stop immediately, allowing for postmortem excitation-induced reactions, referred to as supravital reactions [8]. These reactions include the mechanical and electrical excitability of the skeletal muscle as well as the pharmacologically induced excitability of the iris [8]. According to literature there are two phenomena involving the postmortal mechanical excitability of the muscle, “Zsako phenomenon” (up to 2.5 h postmortem) which involves the quadriceps femoris muscle, and “Idiomuscular contraction (bulgur)” (up to 13 h postmortem) which involves the biceps brachii muscle [5, 14, 15]. The electrical excitability of the skeletal muscles represents an additional method for estimating the time of death. Most studies in this domain examined the excitability of the facial muscles, namely the orbicularis oculi and the orbicularis oris muscle [8]. Based on the investigation from Klein and Klein [16], the pharmacologically induced excitability of the iris allows for a temporal narrowing of the postmortem interval, with reported effective time ranges of approximately 3 to 10 h for atropine, 5 to 30 h for tropicamide (Mydriaticum Roche), and 14 to 46 h for acetylcholine. However, some studies showed that measuring the postmortem pupil size limited robustness regarding the time since death estimation [17, 18].

In practice, the estimation of the time since death is typically performed at the scene. Several methodologies currently exist to estimate the postmortem interval. Traditionally, the estimation can be calculated using the nomogram [4] and the integrating chart for casework [5] and corrective factors [2] with pen and paper. Alternatively, various digital tools, such as websites, mobile applications, and software programs, have been developed to assist in calculating these estimates [19–21]. However, no free-of-charge mobile application currently exists that implements the compound method in a comprehensive and integrated manner. We therefore aimed on developing an application called “ToD” that runs completely offline on iOS and Android systems. It provides a comprehensive on-site solution for estimating the time of death based on the compound method, supported by a user-friendly interface and free of charge.

## Methods

The development of the mobile application followed a structured and iterative approach to ensure accuracy, usability, and efficiency. The process involved selecting an appropriate programming framework, designing a user-friendly interface, implementing robust calculations, and enabling seamless data handling.

### Requirements and application concept

The first step in the development process was gathering user requirements from forensic medical personnel. This step ensured that the application met practical needs and intuitive data input (see Appendix 1). The collected requirements guided the selection of features, data structures, and the user interface design.

### Programming framework

Moreover, based on the project requirements, suitable programming languages and frameworks were chosen. Key criteria included computational capabilities, ease of handling mathematical functions, and cross-platform usability. Hence, Flutter [22] (version 3.22.2) was selected as the development framework due to its cross-platform capabilities, ensuring the application could be deployed on both Android and iOS devices without requiring separate codebases. Written in Dart programming language [23] (version 3.4.3), Flutter provides high performance and a smooth user interface, which is crucial for an application that requires accurate data input and real-time processing. By leveraging Flutter, the application achieved an optimal balance between functionality, user experience, and computational efficiency, making it well-suited for forensic time of death estimation. The development process was managed using the integrated development environment (IDE) Visual Studio Code [24] (version 1.98.2) and version control with GitHub [25] to enable simultaneous collaboration. The workflow followed an iterative approach, allowing incremental improvements and bug fixes.

### Time of death calculations

In our mobile application, the estimation of the time of death based on the compound method was conducted following the standard forensic procedure. We integrated mathematical calculations based on the rectal temperature with corrective factors in combination with rigor mortis, mechanical excitability of the skeletal muscle, electrical excitability of facial muscles and pharmacological excitability of the iris [4, 5, 26, 27].

Temperature-based estimation:

The following equation by Henssge [1, 4, 28] was used to estimate the PMI with the later integration of corrective factors [2]:

1 $$Q=\frac{Tr-Ta}{T_0-Ta}=A\ast e^{Bt}+\left(1-A\right)\ast e^{\left(\frac{A\ast\:B}{A-1}\ast\:t\right)}$$

with

- Q = standardized temperature.
- * Tr* = rectal temperature in Celsius (°C).
- * Ta* = ambient temperature in °C.
- $\:{T}_{0}$= rectal temperature at death (t = 0), assumed to be 37.2 °C.
- t = death time.
- m = body weight in kg.
- A = 1.25 with * Ta* ≤ 23.2 °C or 1.11 with * Ta* ≥ 23.3 °C.
- B = -1.2815 (C * m^-0.625^) + 0.0284.
- C = corrective factor (default = 1), depending on * Ta*:
  - ○ ≤ 23°: Q = $$\:\frac{Tr-Ta}{37.2-Ta}=$$1.25e^Bt^ – 0.25e5^Bt^
  - ○ ≥23°: Q = $$\:\frac{Tr-Ta}{37.2-Ta}$$ = 1.11e^Bt^ – 0.11e^5Bt^

For the estimation of t, a Dart-based numerical script was implemented to iteratively solve the following equation:

2 $$\:\frac{Tr-Ta}{{T}_{0}-Ta}\:\:\sim\:({A}*{{e}}^{Bt}+\left(1-A\right)*{e}^{\left(\frac{A*\:B}{A-1}*\:t\right)})\:$$

The algorithm determines the value of t that minimizes the deviation between the left-hand side and right-hand side of the equation. This process was limited to 1000 iterations.

If the environmental conditions deviate from the standard conditions defined by Henssge, i.e. uncovered body on a thermally indifferent surface within still air, a corrective factor can be applied to account for the deviations, e.g. covered or wind [2, 4]. This corrective factor is already considered in Eq. 1; however, it is based on the body weight. To account for this, Eq. 3 was integrated into the calculation derived from [2, 29] in our application:

3 $$\:F={\left[\:\frac{\frac{-1.2815}{\left(\left({\mathrm{m}}^{-0.625}\right)-0.028\right)\left(-3.24696*{e}^{-0.89959*F70}\right))-0.0354}\:}{m}\right]}^{1.6}$$

- m = bodyweight in kg.
- F^70^ = corrective factor with a bodyweight of 70kg.

To account for uncertainty, the 95% tolerance intervals described by Henssge [4] were incorporated. Specifically, deviations of ± 2.8 h were applied for Q values between 1.0 and 0.5, ± 3.2 h for Q values between 0.5 and 0.3, and ± 4.5 h for Q values between 0.3 and 0.2. When corrective factors were included, the corresponding tolerance intervals were ± 2.8 h, ± 4.5 h, and ± 7.0 h, respectively. For Q values below 0.2, the estimation of the postmortem interval is considered unreliable; therefore, such cases were excluded from calculation within the application and flagged as errors.

Non-temperature-based estimations:

For our purposes, six stages of lividity were considered in relation to estimating the time of death. Only the rigor mortis stages relevant to this estimation were included in the implementation [5], specifically “Beginning,” “Full development,” and “Complete resolution,” as according to Mallach’s statistical analysis [9]. According to Klein et al., the electrical excitability of the facial muscles after the stimulation of the orbicularis oculi muscle can be classified into six grades, allowing a certain delimitation of the time of death [16]. Additionally, the electrical stimulation and reaction of the orbicularis oris muscle provides limitations regarding the time of death [27]. Furthermore, the chemical excitability of the iris was included with caution into the application due to previous findings suggesting that this method is not suitable for forensic time since death estimations [17, 18]. These non-temperature-based estimation methods were integrated into the application. The corresponding details are summarized in Table 1.

**Table 1:** Summary of the non-temperature-based methods to estimate the time of death included in the mobile application, hours post-mortem (hpm)

		Minimal hpm	Maximal hpm	
**Livor mortis** [3, 5, 9]				
Begin	YES	> 0.25	< 3	NO
Confluence	YES	> 1	< 4	NO
Maximum	YES	> 3	< 16	NO
Thumb pressure (complete blanching)	NO	> 1	< 20	YES
Fully redistributed	NO	> 2	< 6	YES
Partly redistributed	NO	> 4	< 24	YES
**Rigor mortis** [9, 13]				
Begin			< 7	NO
Maximum	YES	> 6	< 10	NO
Re-establishment	NO	> 2	< 20	YES
**Electrical excitability of the facial muscles** [5, 7, 16]				
I local upper eye lid	NO	> 5	< 22	YES
II 1/3-2/3 upper eye lid	NO	> 5	< 16	YES
III upper eye lid	NO	> 3.5	< 13	YES
IV upper eye lid + lower eye lid	NO	> 3	< 8	YES
V upper eye lid + lower eye lid + forehead	NO	> 2	< 7	YES
VI upper eye lid + lower eye lid + forehead + cheek	NO	> 1	< 6	YES
Orbicularis oris muscle	NO	> 3	< 8.5	YES
**Mechanical excitability of skeletal muscles** [3, 5, 14, 15]				
Zsako’s muscle phenomenon	NO		< 2.5	YES
Idiomuscular bulg	NO	> 1.5	< 13	YES
**Chemical excitability of the iris** [30]				
Atropin	NO	> 3	< 10	YES
Tropicamide	NO	> 5	< 30	YES
Acetylcholin	NO	> 14	< 46	YES

### Data handling

The system of the mobile application was designed to accept one or multiple types of inputs of postmortem changes for estimating the time of death. In addition, the application was designed to incorporate an error-handling mechanism that evaluates the consistency of the entered parameters and alerts the user when contradictory or implausible input combinations are detected. Features such as editing capabilities and input validation were integrated to improve accuracy and reliability.

### Additional features

To support multilingual functionality, a comprehensive localization strategy was implemented. This was achieved through the integration of the flutter_localizations package, which enables seamless adaptation of the application to multiple languages. The supported languages include English, German, Italian, French, Spanish and Croatian. Furthermore, the application was designed to generate and export results in a standardized document format (PDF). This functionality was facilitated using the pdf library for Dart/Flutter (version 3.11.3), which ensures that essential information is systematically structured and can be exported as a standardized PDF file.

### Evaluation

To verify the reliability of the implementation, the application was tested using multiple input scenarios. A total of 100 previously documented cases, in which the PMI had been estimated using the nomogram and non-temperature-based calculations, were selected. The corresponding input parameters were entered into the application to calculate the PMI interval. To quantitatively assess the agreement between the application-based calculations and the reference values, the intraclass correlation coefficient (ICC 3.1; two-way mixed single measures) analysis was applied. Additionally, a Bland-Altman analysis was conducted to further assess systematic differences and measurement variability. Separate analyses were conducted for the lower and upper limits of the estimated PMI ranges to account for potential variations across the measurement spectrum.

Finally, to enhance usability, the application underwent unsystematic on-site testing by forensic pathologists, who freely used the app during their normal working day. Feedback gathered during this process was used to refine the application where appropriate.

## Results

### Create a new estimation calculation

**Fig. 1:**
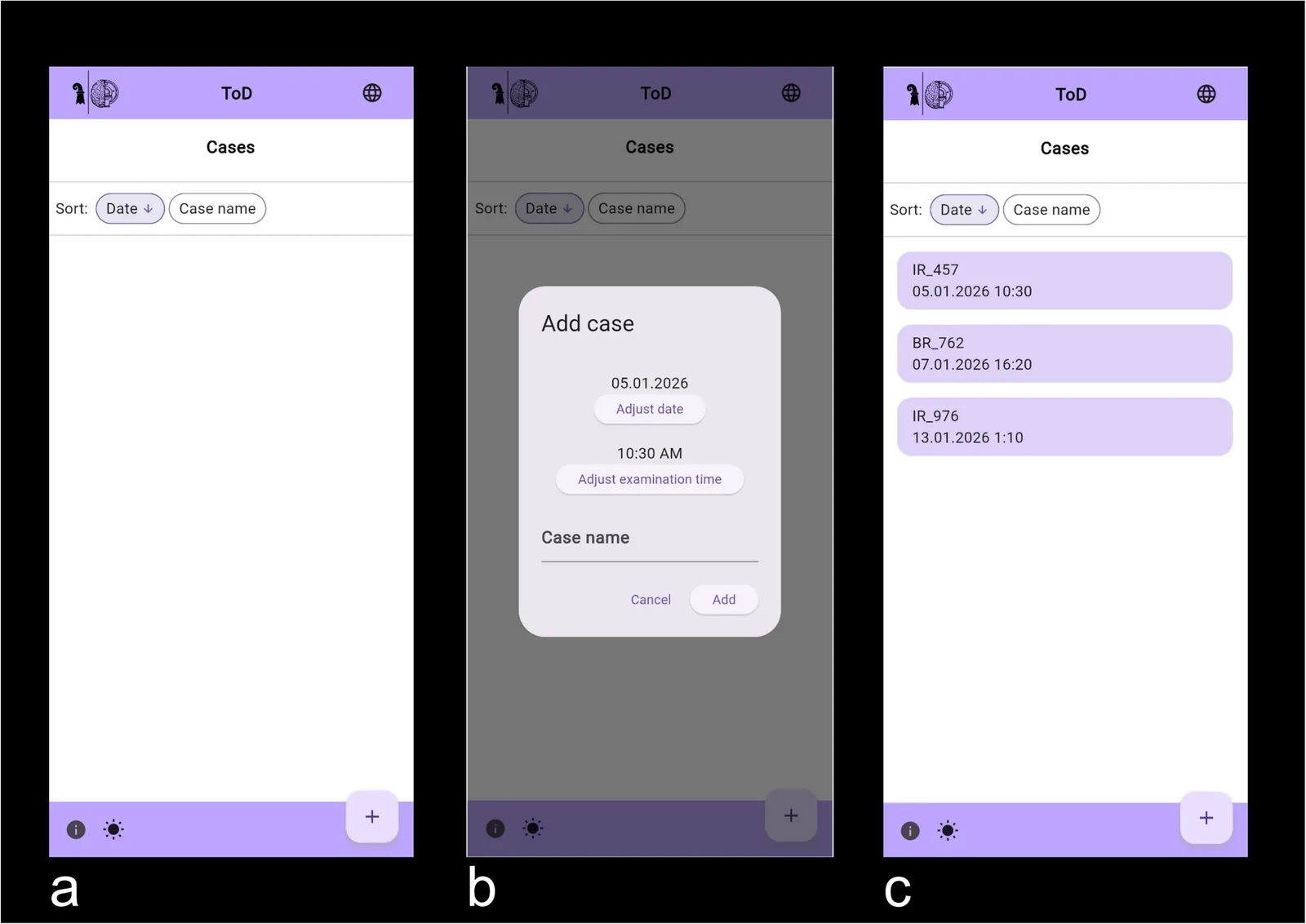
Screenshots showing the home screen of the ToD mobile application: without any cases registered (**a**), during the registration of a new case (**b**), and with newly registered cases (**c**)

Our mobile application named ToD estimates the time of death by analyzing early postmortem changes. The user can assign a unique identification name or number to ensure proper documentation and reference. To comply with privacy and data protection regulations, the naming should not contain any personal identifiable information. Figure 1 displays the home screen of the ToD mobile application along with the process of creating a new case.

A fundamental prerequisite for the accurate estimation of the postmortem interval (PMI) is the precise documentation of the examination date and time. Therefore, the application mandates the input of both parameters at the time of case creation. Since forensic investigations may require modifications or corrections based on additional findings, the recorded date and time of examination remain adjustable.

Once a case is created, all associated data is securely stored locally on the device’s memory, enhancing data security and preventing unauthorized external access. This local storage ensures that the recorded information remains available for subsequent re-evaluation, allowing forensic experts to reassess the input data at any given point in time without relying on external servers or cloud-based storage solutions. Following the creation of a new case, the application enables the input of essential forensic data required for the calculation of the time of death interval, which are explained in the next sections.

### Calculating the time of death

Users can freely select or deselect options according to their needs. Inputs that result in contradictory combinations are automatically detected and displayed as an error message in the user interface. In addition, cases in which a calculation cannot be performed, such as when rectal temperature and environmental temperature are too similar to allow a reliable estimation, are likewise identified and displayed to the user via an error message. Explanatory information is provided at selected points where additional clarification may be beneficial. Furthermore, a reset button allows users to restore the temperature inputs to their default values if they wish to start the process from the beginning.

**Fig. 2:**
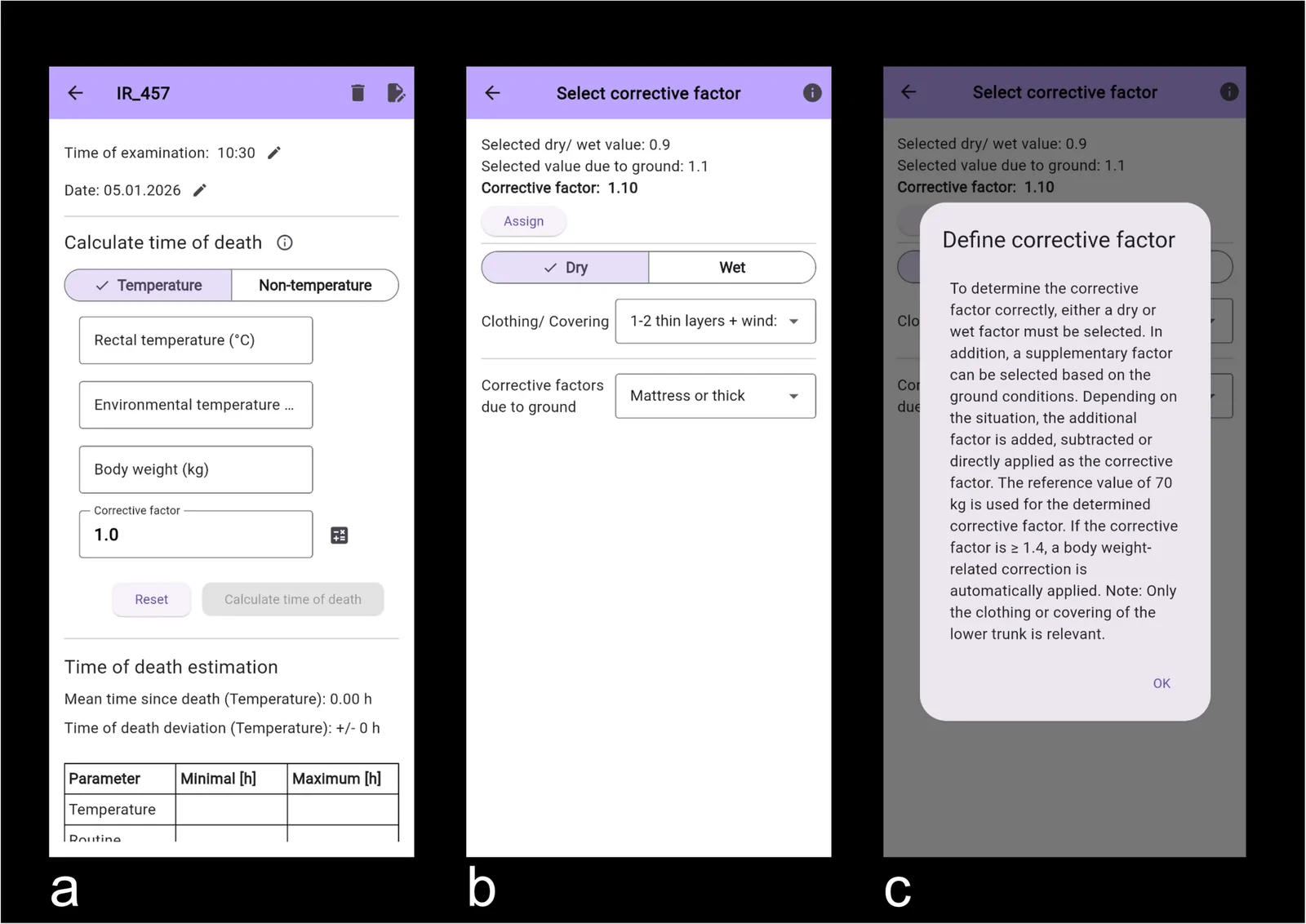
Screenshots displaying the case page of the ToD mobile application: inputting the temperatures (**a**), selecting the corrective factor based on environmental conditions (**b**), explanatory text (**c**)

Temperature-based estimation:

The time of death using the body temperature is estimated based on Henssge, which incorporates corrective factors in the calculation [4, 31]. For this purpose, the body core temperature, environment temperature, body weight, and the corrective factor must be entered. The application allows determination of the relevant corrective factors based on the environment conditions at the scene. The corrective factor can be entered directly or selected based on the environment by clicking the calculator icon next to the correction factor field, as shown in Fig. 2. The corrective factor can be selected based on clothing, surface, and environmental conditions. If the corrective factor exceeds 1.4, a body weight adjustment [29] is automatically calculated and the user is informed that this calculation was integrated. If no corrective factor has been chosen, a default corrective factor of 1.0 is used and has no impact on the calculation. Additionally, the user interface integrates an information pop-up for additional support.

Non-temperature-based estimation:

Similar to the integrating chart for casework at scene [5], the app distinguishes between “Routine” and “Supplement” examinations.

**Fig. 3:**
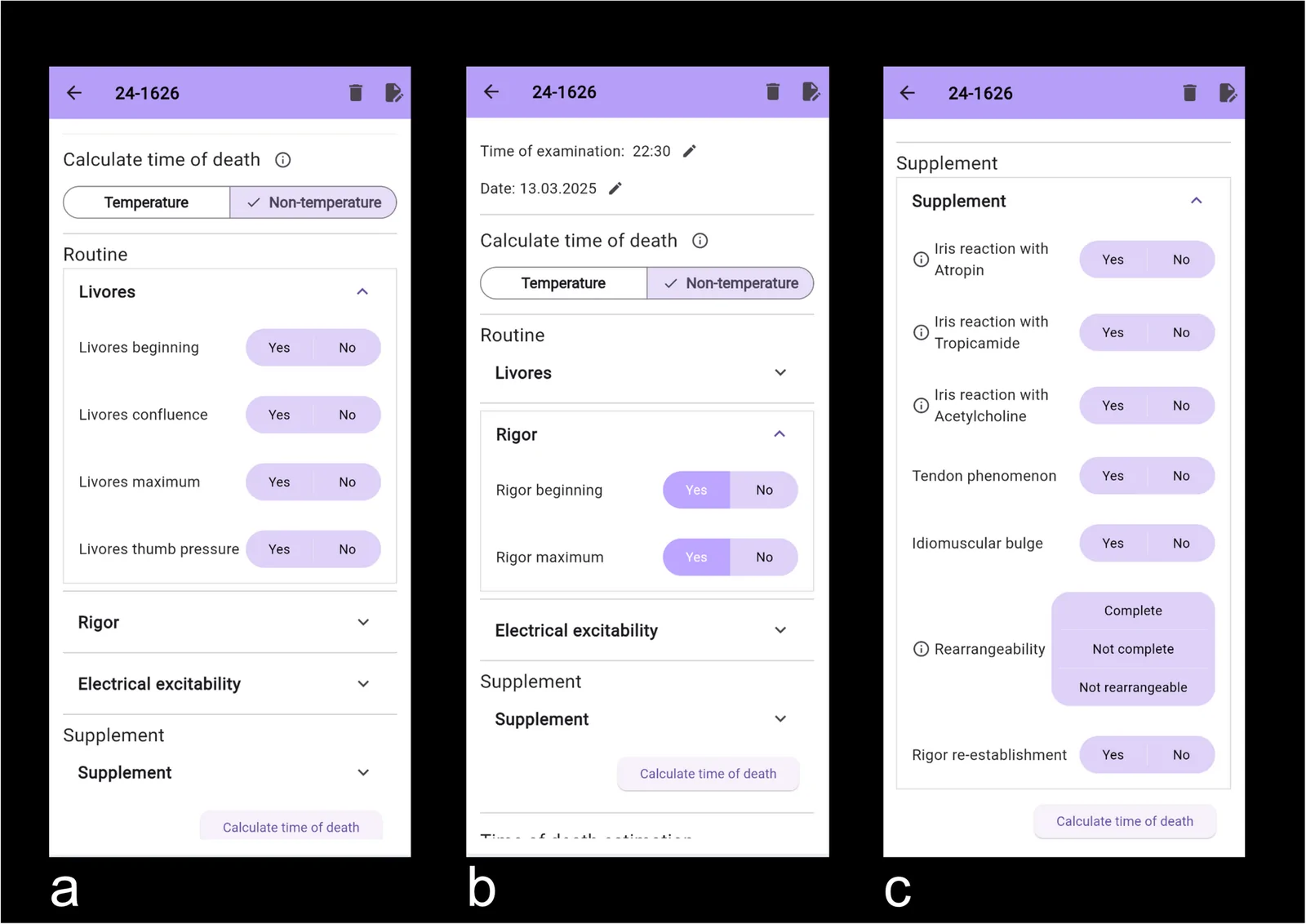
Screenshots showing the case page of the ToD mobile application, where the characteristics of livor mortis (**a**), rigor mortis (**b**) and elements of the supplement category (**c**) can be selected

Within the Routine segment, dropdown menus for “Livores”, “Rigor” and “Electrical excitability” provide structured input options for documenting relevant characteristics (see Fig. 3 for exemplary interface elements). Users can either select between a binary (“YES” or “NO”) response or a multiple-choice response. Options are “Livores Start”, indicating whether livor mortis has begun to develop; “Livores Confluence”, denoting whether hypostatic blood pooling has merged into larger, confluent patches; “Livores Maximum”, specifying whether livor mortis has reached its fullest extent; and “Livores Thumb Pressure”, assessing whether the discoloration completely fades upon the application of localized pressure. Moreover, “Rigor Start”, indicating whether rigor mortis has begun to develop, and “Rigor Maximum”, specifying whether complete rigidity has been achieved. Additionally, the electric dropdown menu allows users to document the electrical excitability of the M. orbicularis oculi by selecting a graded response from I to VI or alternatively choosing “None” if electrical excitability is absent. The reaction for M. orbicularis oris can also be documented with a binary selection.

The supplement section includes the chemical excitability and additional options that work as supplementary input possibilities. The established options for chemical excitability of the iris within the compound methods were added, but with a visual cautionary note since experiments showed misleading results [17]. Additionally, options for mechanical excitability of the M. biceps brachii, allowing users to specify if a reaction of the Tendon Phenomenon or the Idiomuscular Bulge were present. Additionally, for rearrangeability of the corpse, a multiple-choice menu allows the user to specify if the livor mortis remains fully, partially mobile or not rearrangeable at all upon repositioning of the body. For rigor re-establishment, the options allow users to indicate whether rigor reformation has been observed or absent.

These structured data entries ensure the standardized documentation of forensic parameters, allowing for a systematic and reproducible assessment of postmortem changes, thereby enhancing the accuracy and reliability of time of death estimation within our application.

**Fig. 4:**
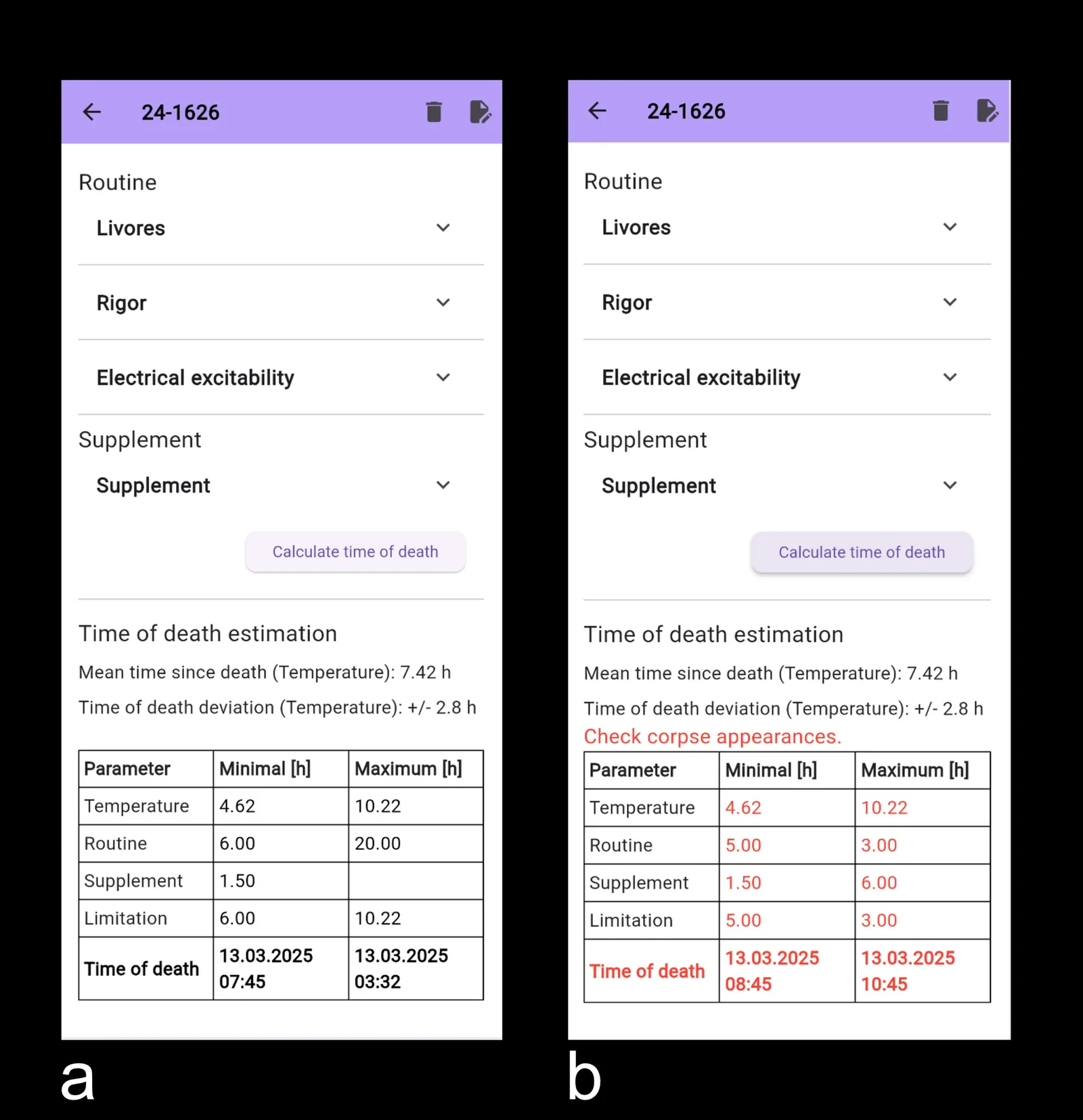
Screenshots showing the calculation of the estimated postmortem interval using all available parameter categories (**a**) and the error prompt if inputs are contradictory (**b**)

The time of death estimation within the application is designed to function dynamically based on the availability of forensic data at the scene. For parameters categorized under “Temperature”, “Routine”, and “Supplement”, the system calculates both a minimum and maximum estimate for the elapsed time since death. Table 1 delineates the specific temporal intervals associated with distinct stages of livor mortis, rigor mortis, the electrical and mechanical excitability of skeletal muscle, and the chemical excitability of the iris, all of which serve as indicators for PMI estimation. Within each parameter category, the lower bound of the estimated interval can only be confirmed or refined by criteria with a higher lower limit, while the upper bound can only be confirmed or refined by criteria with a lower upper limit. In addition to category-specific estimates, the system synthesizes a combined PMI estimate by applying the same constraint-based logic across the categories, thereby generating an interval for the estimated time since death, illustrated in Fig. 4.

### Technical evaluation

The ICC indicated excellent reliability between both methods for the lower (ICC > 0.99) and upper limit (ICC > 0.98), suggesting a very high degree of consistency in the measurements. The Bland-Altman analysis for the lower limit demonstrated a minimal bias (-0.19), indicating a negligible tendency for the standard method to yield slightly lower values. The limits of agreement ranged from − 1.55 h to 1.16 h, showing a narrow dispersion of differences. For the upper limit, the Bland-Altman analysis showed again a small bias of 0.26, indicating minimal systematic differences. The limits of agreement ranged from − 2.42 h to 2.94 h, demonstrating a tight distribution around the mean and no evidence of systematic deviation.

### User experience

The application is designed to enhance user experience through an intuitive and error-resistant interface. If incorrect data is entered or mandatory information is missing, the system generates real-time error messages to alert the user and ensure data integrity. If an incorrect input is provided, the user can negate the selection by re-clicking on the chosen option. Additionally, the application features multilingual support, allowing users to switch between the primary Swiss languages - German, French, and Italian - as well as English, Croatian and Spanish. This functionality ensures accessibility for a diverse user base, facilitating seamless operation across different linguistic regions.

### Sharing the calculation results

**Fig. 5:**
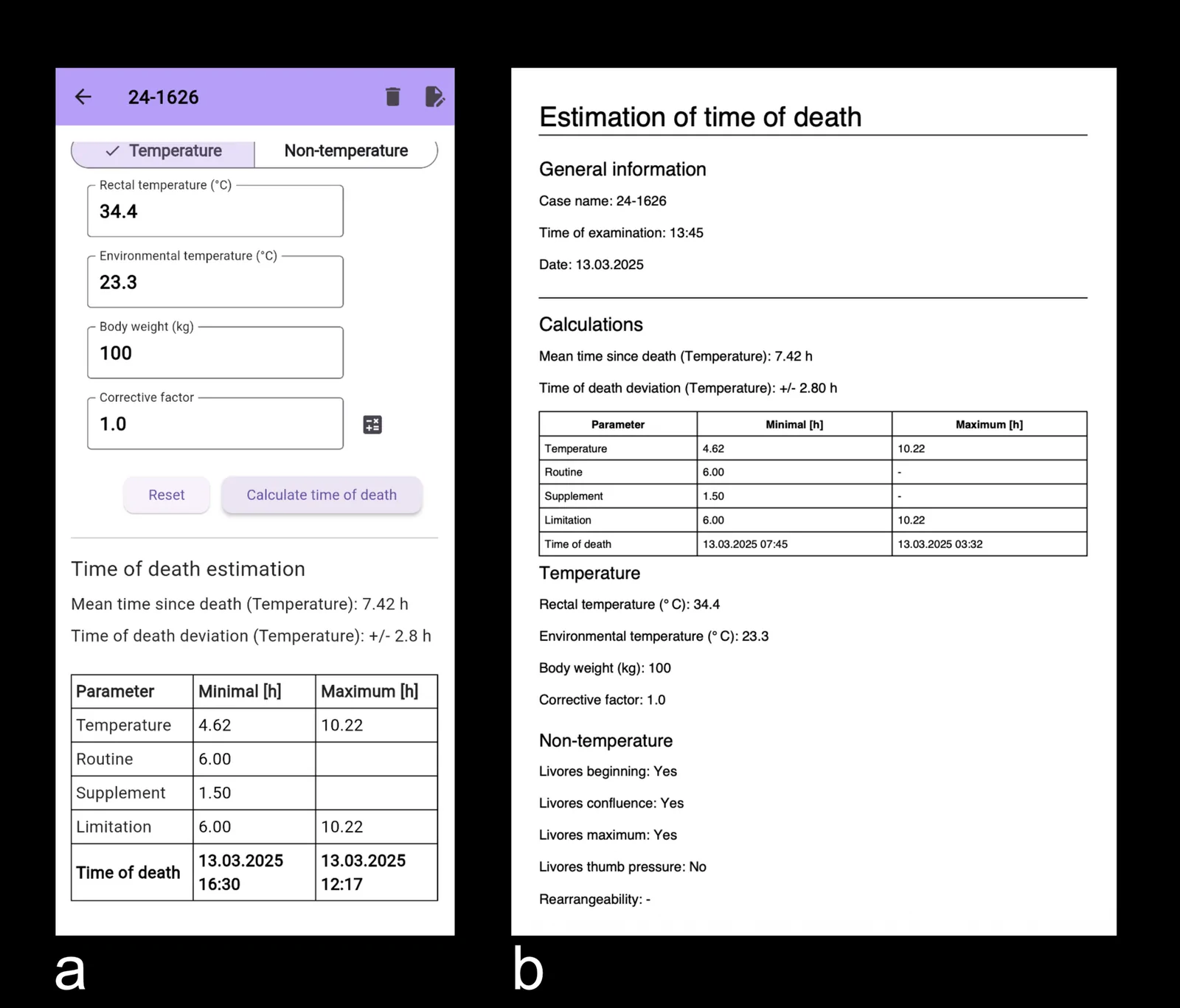
Screenshots displaying the case page of the ToD mobile application, with the resulting time of death estimation shown in the overview table (**a**) and the generated PDF from the case (**b**)

The application includes an export functionality, allowing both the user-inputted data and the calculated time of death estimation to be generated as a PDF report, as illustrated in Fig. 5. This feature enables forensic investigators to securely store case information for documentation purposes or share it when necessary. The generated PDF can be saved locally for record-keeping or integrated into official forensic case files, ensuring a structured and standardized format for reporting.

### App distribution

The application ToD is available for download on both the Apple App Store and Google Play Store, ensuring broad accessibility across iOS and Android devices for forensic professionals. The app is available in the app stores under the name “ToD – estimation”, with the subtitle “Time of death estimation” (Apple App Store: https://apps.apple.com/ch/app/tod/id6744065182?l=de-DE, Google Play Store: https://play.google.com/store/apps/details?id=ch.irm.tod).

## Discussion

The time of death estimation is a crucial part of the forensic investigation. The estimation of an early postmortem interval is based on the compound method by Henssge and Madea [7, 8]. Besides livor mortis, rigor mortis, supravital reactions and pharmacologically induced excitability of the iris, the body core temperature cooling enables the estimation of the time of death [8].

The current gold standard for estimating the time of death based on body core temperature is the Henssge nomogram [4]. Henssge proposed a model for body core temperature cooling under standard conditions, defined as a naked body lying supine on a thermally neutral surface in still air within a closed room without heat sources, which also incorporates corrective factors to estimate the postmortem interval when actual conditions deviate from this standard scenario [4].

In current forensic practice, the time of death estimation is performed manually on-site. This manual calculation can complicate the execution with the Henssge nomogram due to limited practical capabilities at the scene (e.g., darkness, rain, or the absence of a suitable surface for pen-and-paper calculations). Similarly, software-based approaches may also be challenging because using a laptop can be difficult under such conditions and internet access may be limited. Our mobile application facilitates rapid and reliable on-site calculation by enabling simple, one-handed data entry and functioning independently of internet connectivity.

Several software solutions exist to support postmortem interval estimation, each with different capabilities, as shown in Table 2. The software provided by AMASoft [20] integrates the compound method with corrective factors and operates offline; however, it is only available for Windows systems and the full version is not free of charge, which limits accessibility and mobile use. The browser-based calculator available on SwissWuff [21] supports mobile devices and is free of charge, but it requires an internet connection and only provides temperature-based calculations without export functionality. The iTOD App [19] allows temperature-based estimations alongside additional calculations using other factors and can perform calculations offline on mobile devices. However, it only supports English and export functionality is limited to email. In contrast, the mobile application presented in this study integrates the compound method with automated adjustment of corrective factors while remaining freely available

**Table 2:** Comparison of the available digital tools to estimate the time of death

Feature	AMASoft	SwissWuff calculator	iTOD app	ToD application
Temperature-based calculation	Yes	Yes	Yes	Yes
Compound method	Yes	No	No	Yes
Offline functionality	Yes	No	Yes	Yes
Mobile support	No (Windows only)	Yes (browser)	Yes	Yes
Export functionality	PDF export	No	Email only	PDF export
Cost	Not fully free	Free	Free	Free
Languages	German, English	German, English	English	Multiple languages
Additional usability features	Explanatory guidance	-	Explanatory guidance	Explanatory guidance, dark/light mode

When comparing the standard method (Nomogram with case worksheet) with the application-based implementation, the results demonstrate overall excellent agreement, supporting the validity of the application-based calculations. Some deviations were noted in temperature-based calculations, likely due to routine rounding of time values to 15-minute intervals at our institute, which can affect calculations dependent on precise timing. However, both the ICC > 0.99 and the Bland–Altman analyses indicate excellent reliability and minimal systematic bias. Therefore, the two methods can be considered highly consistent and generally comparable, supporting the use of the application-based approach as an alternative to the standard method.

In addition to the computational power to estimate the time of death, our mobile application also automatically identifies any inconsistencies in the entered data. This feature helps detecting inconsistencies on-site, allowing for a direct review and reassessment of the postmortem changes. Our application further operates offline on mobile devices (iOS and Android), provides export functionality in PDF format, and supports multiple languages. Additionally, usability features such as dark and light mode and explanatory guidance were implemented to facilitate practical application in forensic casework.

Accurate determination of the post-mortem interval remains a significant challenge in forensic medicine [32–34]. Methods such as immunohistochemical and histological analyses [37], as well as the incorporation of physiological parameters including body mass index and body surface area, have been proposed to improve the predictive accuracy of time-since-death estimations [35]. In addition, several studies have investigated the use of potassium concentration in the vitreous humor as a biochemical marker for estimating the PMI, providing a complementary approach to the Henssge method [35]. Furthermore, emerging approaches, particularly metabolomics combined with machine-learning techniques for post-mortem intervals exceeding 72 h, represent promising avenues of research [36, 37]. However, the reliability of any predictive tool or digital application ultimately depends on the quality and validity of the established ground truth on which it is based. Defining and validating such reference standards therefore remains a fundamental challenge. Future developments of our mobile application are intended to incorporate emerging research findings that may further enhance its accuracy and practical relevance.

## Conclusion

Our mobile application ToD was developed to facilitate on-site time of death estimation based on the compound method, addressing the limitations of traditional manual methods. Designed to assist forensic pathologists during investigations, the app provides a more convenient approach to postmortem interval assessment in the field. Built using the Flutter framework, the application ensures platform independence, allowing deployment on both iOS and Android devices. Its integrated calculation engine operates entirely offline, eliminating the need for an internet connection while delivering rapid and reliable time of death estimates. This design guarantees that forensic professionals can perform critical computations in real-time with consistent performance.

## Appendix 1

### Requirements and usability

Requirements from our medical personnel:

- Estimation integrates compound method based on Henssge.
- Works on mobile phone and tablets, on Android and iOS operating systems.
- Calculations works offline.
- Supports various languages.
- Export functionality.
- Case handling: creation, edit (date and time, all parameters to calculate the PMI), delete.
- Must be intuitively useable without an instruction document.
- Handles error-handling when user inputs data that is contradictory.
- Is free of charge.
